# The human gut resistome

**DOI:** 10.1098/rstb.2014.0087

**Published:** 2015-06-05

**Authors:** Willem van Schaik

**Affiliations:** Department of Medical Microbiology, University Medical Center Utrecht, 3584 CX Utrecht, The Netherlands

**Keywords:** antibiotic resistance, gut microbiota, metagenomics, gut commensals, *Enterococcus faecium*

## Abstract

In recent decades, the emergence and spread of antibiotic resistance among bacterial pathogens has become a major threat to public health. Bacteria can acquire antibiotic resistance genes by the mobilization and transfer of resistance genes from a donor strain. The human gut contains a densely populated microbial ecosystem, termed the gut microbiota, which offers ample opportunities for the horizontal transfer of genetic material, including antibiotic resistance genes. Recent technological advances allow microbiota-wide studies into the diversity and dynamics of the antibiotic resistance genes that are harboured by the gut microbiota (‘the gut resistome’). Genes conferring resistance to antibiotics are ubiquitously present among the gut microbiota of humans and most resistance genes are harboured by strictly anaerobic gut commensals. The horizontal transfer of genetic material, including antibiotic resistance genes, through conjugation and transduction is a frequent event in the gut microbiota, but mostly involves non-pathogenic gut commensals as these dominate the microbiota of healthy individuals. Resistance gene transfer from commensals to gut-dwelling opportunistic pathogens appears to be a relatively rare event but may contribute to the emergence of multi-drug resistant strains, as is illustrated by the vancomycin resistance determinants that are shared by anaerobic gut commensals and the nosocomial pathogen *Enterococcus faecium*.

## 1. The gut microbiota: a complex ecosystem and a reservoir for antibiotic resistance genes

Antibiotics have become a cornerstone of medicine in the decades since the Second World War, but even before humans initiated the industrial production and widespread use of antibiotics, they have existed in nature for hundreds of millions of years [1]. In natural environments, antibiotics may incapacitate bacteria that compete for scarce resources and thus provide a selective benefit for the producing strain. In addition, antibiotics could function as signalling molecules which may trigger bacterial developmental processes, such as biofilm formation, that contribute to survival [2,3]. Resistance to antibiotics may arise in a population of susceptible bacteria by the accumulation of mutations (e.g. point mutations in DNA gyrase conferring resistance to quinolones) or by the acquisition of resistance genes that protect the cell against antibiotics. Antibiotic resistance genes can cause phenotypic resistance through a variety of mechanisms, including the enzymatic inactivation of the antibiotic, the modification of the antibiotic target and the prevention of the accumulation of lethal intracellular concentrations of the antibiotic through efflux pumps [4,5]. Just as antibiotics have been present in the environment for aeons, antibiotic resistance genes are ancient too, as illustrated by the estimated emergence of the serine β-lactamases over 2 billion years ago [6]. Horizontal transfer of antibiotic resistance genes also pre-dates the human use of antibiotics, as OXA-type β-lactamases have already been carried on plasmids and have moved between bacterial phyla for millions of years [7]. It should be noted, however, that a gene that confers antibiotic resistance may have an entirely different function in its original bacterial host, as is illustrated by the 2′-*N*-acetyltransferase encoding gene in the Gammaproteobacterium *Providencia stuartii*. This enzyme is involved in the modification of peptidoglycan, but because aminoglycoside antibiotics are structurally similar to the natural substrate of 2′-*N*-acetyltransferase, the enzyme can also inactivate aminoglycosides, providing intrinsic resistance to this class of antibiotics to *P. stuartii* [8]. Only when such ‘accidental resistance genes’ are mobilized and transferred to other bacterial hosts, can they contribute to the proliferation of antibiotic resistant human pathogens [3]. Currently, antibiotic resistance among human pathogens has become a major threat to modern medicine and there is considerable interest to identify the niches in which bacteria can gain antibiotic resistance genes and the mechanisms by which horizontal transfer of resistance genes occur.

The human body is populated by an estimated 10^14^ bacteria, including harmless symbionts, commensals and opportunistic pathogens [9]. Different body habitats exhibit differences in bacterial composition, presumably reflecting the different micro-environments of the human body [10]. The human gastrointestinal tract harbours a large and diverse bacteria population, which has an important role in human health and disease. The number of bacteria varies along the length of the gastrointestinal tract, ranging from less than 10^3^ bacteria ml^−1^ in the stomach and the duodenum, increasing to 10^4^–10^7^ bacteria ml^−1^ in the jejunum and ileum. The highest bacterial load is reached in the colon where 10^11^–10^12^ bacteria ml^−1^ are present [9]. The human gut thus harbours a complex microbial ecosystem, which consists of hundreds of species, collectively termed the gut microbiota. The gut microbiota is relatively stable in healthy adults but the composition of the gut microbiota can change rapidly owing to dietary changes, illness and the use of antibiotics [11,12].

The large majority of the bacteria that populate the human gut are strictly anaerobic. Two phyla, the Bacteroidetes and Firmicutes, commonly dominate the gut microbiota of healthy adults. Bacteria from these phyla perform functions that are important for the host, such as the production of vitamins and the degradation of complex carbohydrates from the diet. In addition, several facultatively anaerobic bacteria, like those from the families Enterobacteriaceae and Enterococcaceae are ubiquitous members of the human gut microbiota, but generally at levels that are considerably (at least 100-fold) lower than those of the strictly anaerobic gut commensals [13,14]. The Enterobacteriaceae and Enterococcaceae are of particular interest because organisms from these groups, such as *Escherichia coli*, *Klebsiella pneumoniae*, *Enterococcus faecalis* and *Enterococcus faecium* have emerged as multi-drug resistant nosocomial pathogens of major importance in the last few decades [15,16]. Evidently, the gut can serve as a reservoir for opportunistic pathogens, which may cause infections in immunocompromised individuals. The gut's role as a source of opportunistic pathogens is particularly relevant for hospitalised patients, which are at high risk of developing infections. The large quantities of antibiotics that are used in the treatment of this patient group may select for multi-drug resistant opportunistic pathogens in the gut microbiota. Opportunistic pathogens from the gut may cause infections by translocating across the intestinal barrier or, after faecal contamination of skin and other body sites, may cause infections upon placement of a catheter or an intravenous line [15,17].

Because bacterial populations in the human gut are large and share a similar ecology, there is ample opportunity for the transfer of genetic material [18]. Consequently, there is considerable interest to characterize the antibiotic resistance gene reservoir (‘the resistome’) of the human gut microbiota and to understand to what extent the antibiotic resistance genes can spread between different members of the gut microbiota, particularly between commensals and opportunistic pathogens [19]. In this review, I will highlight recent studies that have used metagenomic approaches to identify and quantify antibiotic resistance genes in the bacteria that populate the human gut. The mechanisms and extent by which antibiotic resistance genes that are harboured by anaerobic commensals can transfer to opportunistic pathogens are also discussed.

## 2. Methods to study the human gut resistome

**Figure 1.**
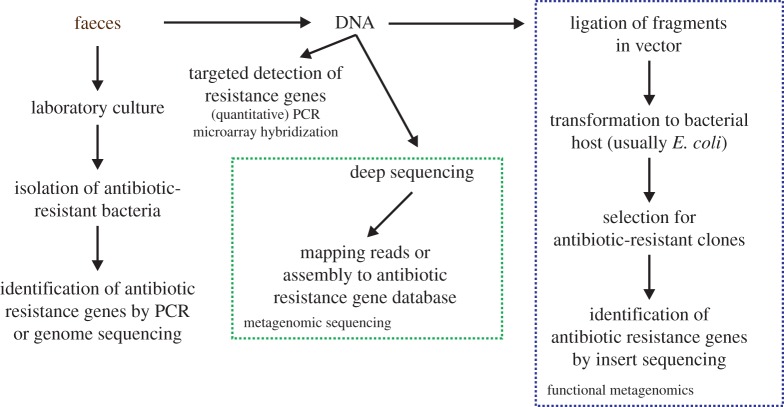
Methods for the analysis of the human gut resistome. Starting from a faecal sample, the resistome can be sampled by culture, through targeted detection of resistance genes (by polymerase chain reaction (PCR) or microarray hybridization), by metagenomic sequencing and by functional metagenomics. Further details of these methods are discussed in the text.

Methodological approaches for the study of the resistome are outlined in figure 1 and are further discussed below. Notably, all current methods to describe the human gut resistome in terms of type and abundance of antibiotic resistance genes are by themselves inadequate to fully characterize the reservoir of resistance genes and the genetic determinants that are associated with the resistance genes. Therefore, a combination of different methodologies should be used if one wants to fully characterize the human gut resistome.

Bacteria from the human gut can be analysed through laboratory culture and subsequent characterization of the isolated strains. Culture-based studies from the 1970s and 1980s have shown that anaerobic gut commensals from the orders Bacteroidales and Clostridiales can carry antibiotic resistance determinants. These bacteria can not only transfer antibiotic resistance genes *in vitro* to closely related bacteria, but also to opportunistic pathogens [20–23]. Nevertheless, the isolation and laboratory culture of gut bacteria has long been believed to be practically impossible [24] and consequently there is a lack of information on the antibiotic resistance genes in gut commensals and whether these genes are linked to mobile genetic elements. Recently, important advances have been reported in the laboratory culture of a wide variety of gut commensals [25–27], which has opened up the possibility of performing culture-based analyses of antibiotic resistant bacteria from the gut microbiota. Comparative genomic studies of intestinal bacteria that serve as reservoirs of antibiotic resistance genes have been performed for the Bacteroidales [28] and Enterococcaceae [29,30]. However, culture-based studies to comprehensively map and characterize the bacteria that carry antibiotic resistance genes in the human gut still remain to be performed and it is as yet unclear whether culture-based methodologies can truly capture the entire complement of antibiotic-resistant bacteria in the human gut.

In addition to laboratory culture, several culture-independent approaches exist that can be used to probe the antibiotic resistance gene reservoir of the gut microbiota by using DNA that is isolated from faecal samples (figure 1). Resistance genes from the gut microbiota can be detected and quantified by quantitative PCR [31,32] or by microarray hybridisation [33,34]. These methods provide a relatively fast overview of the presence and abundance of the targeted antibiotic resistance genes, but they are limited in their ability to detect genes of which the sequence is not fully complementary to the primers and probes and they will, by definition, not provide information on the presence of antibiotic resistance genes that are not targeted by the primer pairs or probes. In addition, PCR and microarray methodologies do not provide information on the genetic context of the resistance genes or the bacterial hosts of the resistance genes.

In an approach termed metagenomic sequencing, DNA that is purified from faeces is sequenced using modern sequencing technologies. The resulting sequencing datasets can be analysed by assembly of the short reads into larger contiguous DNA fragments or by mapping the sequence reads to reference sequences. This method allows the determination of the phylogenetic composition of the microbiota and can be used to simultaneously detect and quantify antibiotic resistance genes in the microbiota [35].

In an alternative approach, termed functional metagenomics, faecal DNA is randomly cloned into an *E. coli* vector, either as less than 5 kbp fragments in a routine cloning vector or as fragments of approximately 40 kbp in a fosmid vector. The libraries of randomly cloned DNA are then plated on media that contain antibiotics, resulting in the isolation of antibiotic-resistant clones from the libraries. The vector inserts of resistant clones are then sequenced to identify the genes that confer resistance to the antibiotic of interest [4,36]. In the case of fosmid libraries, the antibiotic resistance gene that is harboured by a particular fosmid clone can be identified by *in vitro* transposon mutagenesis of the fosmid. Subsequent screening for transposon mutants that have lost the antibiotic-resistant phenotype and sequencing of the transposon insertion sites results in the experimental validation of a gene's function in antibiotic resistance [32,37]. Functional metagenomic analyses of the gut resistome are considerably more labour-intensive then the previously discussed methods but have the advantage that novel resistance genes (i.e. those that are currently not included in antibiotic resistance gene databases) are identified and that, in the case of large-insert fosmid libraries, information can be obtained about the genetic context of antibiotic resistance genes.

## 3. Metagenomics of the human gut resistome

Several studies have recently applied metagenomic sequencing and functional metagenomics to probe the resistome in the gut of healthy [38–41] and hospitalized individuals [32,42].

These studies have revealed that the human gut microbiota forms a large reservoir of antibiotic resistance genes, with Forslund *et al.* [38] finding resistance genes for 50 of 68 classes of antibiotics in 252 faecal metagenomes, at an average of 21 antibiotic resistance genes per sample. These samples were collected from individuals in Spain, Italy, Denmark, France, Malawi, the USA and Japan. Hu *et al.* [39] identified a total of 1093 antibiotic resistance genes in 162 individuals from China, Denmark and Spain, in a dataset that partially overlapped with that analysed by Forslund *et al.* [38]. Genes providing resistance to the antibiotic tetracycline (*tet32*, *tet40*, *tetO*, *tetQ* and *tetW*) are present in the microbiota of all individuals and are also the most abundant family of resistance genes [38,39]. The *tetQ* gene is the most abundant resistance gene in Chinese, Danish and Spanish individuals, which may be explained by the high prevalence of this resistance gene in *Bacteroides* isolates, which has increased from 30% in the early 1970s to more than 80% at the start of the twenty-first century [43]. Other resistance genes that were ubiquitously present putatively confer resistance to aminoglycosides (*ant*(*6′*)*-Ia*), bacitracin (*bacA*) and the glycopeptide vancomycin (*vanRA* and *vanRG*) [39]. However, it should be noted that the Antibiotic Resistance Genes Database (ARDB) [44], which was employed by the authors of both studies, contains many genes that have an unclear role in antibiotic resistance. Specifically, the *bacA* gene appears to be present in most bacterial genomes, where it has a role in peptidoglycan synthesis [45]. In addition, the *vanRA* and *vanRG* genes are involved in the transcriptional regulation of vancomycin resistance genes in enterococci [46]. These regulatory genes are therefore not directly involved in the remodelling of peptidoglycan cross-links, leading to vancomycin resistance, but may be broadly present, regulating genes that do not have a role in vancomycin resistance [47]. The large number of genes with housekeeping or regulatory functions in ARDB should lead researchers to use more recently developed tools and resistance gene databases to characterise the resistome in natural environments, such as CARD [48], RED-DB (http://www.fibim.unisi.it/REDDB), ResFinder [49], ARG-ANNOT [50] or Resfams [51].

Both previously discussed large-scale metagenomic sequencing studies of the gut resistome [38,39] analysed datasets of healthy individuals from different countries, which had very different practices in terms of antibiotic use in human and veterinary medicine. Interestingly, individuals from countries with relatively reticent policies of antibiotic use in humans and animals (specifically Denmark in these studies) have lower levels of antibiotic resistance genes in their gut microbiota than people from countries where antibiotic use is considerably higher, like Spain and China [38]. This finding suggests that policies concerning antibiotic use in human and veterinary medicine can have a major effect on the relative abundance of antibiotic resistance genes in the gut microbiota of the inhabitants of the different countries [38,39]. However, it should be noted that the difference in antibiotic resistance gene abundance in the microbiota of individuals from these countries is relatively small (approx. 1.5 to twofold) and it remains to be determined whether this difference in abundance contributes to the burden of antibiotic resistant infections in these countries. Interestingly, analysis of single nucleotide polymorphisms (SNPs) in the antibiotic resistance genes indicated that the resistance genes have a specific geographical signature, with the sequences of resistance genes from Chinese individuals forming a cluster that is distinct from resistance genes of Danish and Spanish individuals, which are intermingled in a phylogenetic analysis [39]. A similar specific clustering of Chinese individuals was observed when the abundance of resistance genes in different populations was analysed [52]. The regional differentiation in the sequence and abundance of resistance genes again may be linked by the differences in antibiotic use and exposure of the microbiota to antibiotics in China and European countries, but further analyses with additional data from individuals in other, particularly non-European, countries, will need to be performed to confirm these observations.

**Figure 2.**
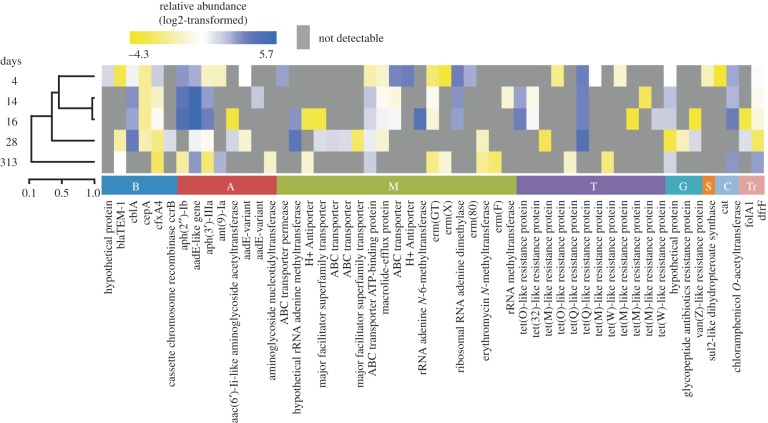
Resistome dynamics of a hospitalized patient. Metagenomic sequencing was performed on DNA that was isolated from faecal samples of a patient that was hospitalized in an ICU (days 4, 14 and 16). After ICU discharge at day 17, further faecal samples were collected during hospitalization in a medium-care ward (day 28) and 270 days after hospital discharge (day 313). The relative abundance of each resistance gene is indicated. Letter codes indicate resistance gene families (B, β-lactams; A, aminoglycosides; M, macrolides; T, tetracyclines; G, glycopeptides; S, sulfonamides; C, chloramphenicols; Tr, trimethoprim). Adapted from [32] with permission from Oxford University Press.

Recently, metagenomic sequencing has been used to study the gut resistome of patients throughout hospitalization [32,42]. As in the studies discussed above, many antibiotic resistance genes were found to be present in the microbiota of the studied patients. The relative abundance of antibiotic resistance genes generally appeared to increase in response to antibiotic therapy. For example, Buelow *et al.* [32] found that the relative abundance of genes that confer resistance to aminoglycosides expanded during hospital stay, particularly during hospitalization in an intensive care unit (ICU) (figure 2). The expansion of the resistome may have been linked to the use of tobramycin (an aminoglycoside antibiotic) that is used as part of a prophylactic antibiotic therapy, termed selective decontamination of the digestive tract, which is used in some European countries to lower the risk of infections with opportunistic pathogens during ICU hospitalization [53]. Similar effects on the resistome were observed in a metagenomic study in which four patients received different courses of antibiotics [42]. However, the use of antibiotics does not always lead to an expansion of the gut resistome in patients and resistance genes from the human microbiota may even be eradicated during antibiotic therapy [32,42]. This can occur during combination therapy, when two (or more) antibiotics are used simultaneously: when a bacterium carries a resistance gene for one of the antibiotics, but is still susceptible to the other antibiotic that is used during therapy, the resistance gene will be lost from the microbial population.

Even though some information on the genetic context of resistance genes can be gleaned by assembling metagenomic sequencing data [52], the bacterial host of antibiotic resistance genes identified by metagenomic sequencing can frequently not be determined with certainty as resistance genes are often associated with promiscuous genetic elements that can be located in a wide variety of organisms. The identification and quantification of antibiotic resistance genes by metagenomic sequencing will also fail to identify genes that have not previously been linked to antibiotic resistance and are therefore not present in antibiotic resistance gene databases that are used to analyse metagenomic sequencing data. Several functional metagenomic studies have been performed to study the human gut resistome and these have provided an important complement to metagenomic sequencing studies. Sommer *et al.* [41] describe a functional metagenomic study of the resistome in faeces and saliva of two healthy individuals. DNA fragments (1–3 kb in size) were randomly cloned into an *E. coli* cloning vector after which the resulting library was screened for phenotypic resistance to thirteen antibiotics, leading to the isolation of 290 antibiotic resistant clones. Subsequently, the plasmid inserts of resistant clones were sequenced to identify the genes that conferred resistance. Interestingly, most of the antibiotic resistance genes that were identified in this way had relatively low nucleotide identity (69.5% on average) to genes that had been deposited at GenBank at the time of analysis. Sequencing of the inserts that originated from faecal material indicated that most of the genes originated from strictly anaerobic gut commensals, such as bacteria from the genera *Bifidobacterium* and *Bacteroides*. Notably, these resistance genes were distinct (on average 60.7% nucleotide identity) from the resistance genes that were found in pathogenic bacteria that can grow aerobically, suggesting that resistance genes from human gut commensals are unlikely to transfer frequently to pathogens. A recent, methodologically similar study found that the resistome of 22 healthy infants and children is already highly diverse and contains genes conferring resistance to 14 of the 18 tested classes of antibiotics [54]. Both novel and previously described resistance genes were identified in the infant gut microbiota and clinically relevant antibiotic resistance genes could be identified, even in the absence of selective pressure in the form of antibiotic therapy.

Because libraries with relatively small inserts were generated in the studies discussed above, only limited information can be obtained on the genetic context of the resistance gene. This limitation can be circumvented by the generation of long-insert (fosmid) libraries of which the inserts can be sequenced in their entirety to allow reliable determination of the origin of the cloned resistance genes and to assess its potential association with mobile genetic elements. Compared to small-insert libraries, fosmid libraries may result in lower numbers of antibiotic resistant clones, because the antibiotic resistance genes have to be expressed from their native promoters rather than a strong, *E. coli-*optimized, promoter in small-insert libraries [55].

Reflecting the high abundance of tetracycline resistance genes in the gut resistome, fosmid clones that are resistant to this antibiotic can be readily isolated from fosmid libraries constructed with faecal DNA. In a study that used fosmid libraries to determine the diversity of tetracycline resistance genes and the bacteria that carry these genes in a mother and her infant child, *tetM* and *tetL* genes were found to be carried by streptococci in the infant metagenomic library [40]. The tetracycline resistance genes *tetO* and *tetW* could only be detected by PCR in the uncloned DNA that was purified from the infant faecal sample. By contrast, *tetM* and *tetL* were not found in the metagenomic library of the mother. Instead, this library contained the tetracycline resistance genes *tetO*, *tetW* and *tetX*, which were carried by strictly anaerobic Bacteroidetes and Firmicutes [40]. This observation suggests that resistance gene transfer can occur from the maternal microbiota to the child. However, skin bacteria (including streptococci and staphylococci) which colonize the infant intestinal tract in the first days and weeks of life are likely to be more important sources of antibiotic resistance genes [56].

Buelow *et al.* [32] have applied fosmid-based functional metagenomics, in addition to metagenomic sequencing, to map the gut resistome during and after patient hospitalization. The authors were able to isolate five clones that acquired resistance to the β-lactam ampicillin, the macrolide erythromycin and tetracycline. Antibiotic resistance genes in the fosmid were identified by *in vitro* transposon mutagenesis and all fosmid inserts were sequenced to completion, allowing the analysis of the genetic context of the resistance gene. All resistance genes were predicted to originate from anaerobic gut commensals (Bacteroidets, Firmicutes and Actinobacteria). Notably, the aminoglycoside resistance *aph(2″)-Ib*, which was predicted to originate from the Firmicute *Subdoligranulum*, was genetically linked to genes with putative roles in plasmid conjugation and replication. This gene has previously been described in the opportunistic pathogen *E. faecium* and may contribute to high-level aminoglycoside resistance in this organism [57].

Metagenomic studies have mapped the human gut resistome in great detail and have shown that anaerobic gut commensals form the main reservoir of antibiotic resistance genes. However, many questions remain on the potential mechanisms by which resistance genes can circulate among the bacteria in the gut microbiota and the extent by which these can transfer from mostly harmless gut commensals to opportunistic pathogens.

## 4. Gut commensals as hubs for resistance gene transfer in the gut

**Figure 3.**
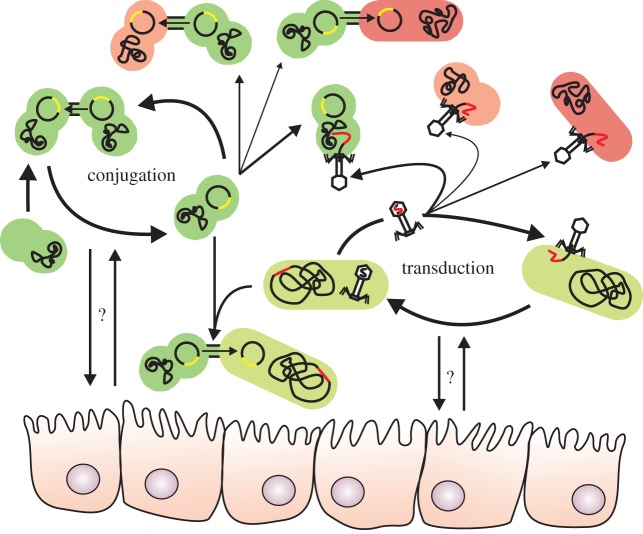
A schematic overview of the pathways for horizontal gene transfer in the human gut. Anaerobic commensal bacteria (green) form an important reservoir of antibiotic resistance genes. Horizontal gene transfer owing to conjugation (illustrated by plasmid transfer) and bacteriophage-mediated transduction appears to be a common event, particularly between closely related bacteria. Gut-dwelling opportunistic pathogens (red), such as the Enterobacteriaceae and the Enterococcaceae, can also acquire resistance genes from the gut microbiota, but this is likely to occur at lower rates, which may be due to their small population size compared with the anaerobic gut commensals. The relative contribution of the different mechanisms for horizontal gene transfer of antibiotic resistance genes from gut commensals to opportunistic pathogens remains to be determined, but gene transfer may be more common between bacteria from the same phylum (like the Firmicutes) than between bacteria from different phyla. The role of the host, as illustrated by the epithelial cells (not drawn to scale), in mediating gut conditions that may positively or negatively affect horizontal gene transfer has only recently been recognized and also deserves further study.

Horizontal transfer of antibiotic resistance genes can occur through several different mechanisms (figure 3). During transformation, naked DNA, which may contain antibiotic resistance genes, is taken up by a bacterium. During conjugative transfer, resistance genes can spread after the formation of a mating bridge between two cells, via which plasmids or conjugative transposons can move from a donor cell to a recipient. In transduction, resistance genes that are encoded by bacteriophages can transfer from one cell to another and be integrated into the chromosome of the recipient cell, in a process called lysogeny [58]. Of these three processes, transformation does not appear to contribute appreciably to horizontal gene transfer in the mammalian intestinal tract [59], but both conjugation and transduction importantly contribute to the spread of antibiotic resistance genes in the gut microbiota.

Conjugation of plasmids or conjugative transposon between related organisms appears to be a relatively common event in the gut [28,30,60] and conjugation may therefore contribute importantly to the spread of resistance genes. Analyses of genome and plasmid sequences have indicated that conjugation between remotely related bacteria, while rarer than between closely related bacteria, contributes to the dissemination of antibiotic resistance genes [61,62]. Further evidence for *in vivo* conjugative spread of resistance genes from anaerobic gut commensals to opportunistic pathogens is illustrated by the observation that the *vanB*-type vancomycin resistance transposon is commonly carried by anaerobic gut commensals of the phylum Firmicutes [63,64], which can serve as a continuous source for vancomycin resistance genes for the nosocomial pathogen *E. faecium* [65]. Among Gram-negative bacteria, the Bacteroidetes appear to play an important role in the *in vivo* transfer of resistance genes as they are able to successfully acquire DNA from a wide variety of bacteria, including the Gram-positives *E. faecalis* and *Clostridium perfringens*, which can then be transferred to other gut dwelling bacteria [43]. A specific bacteroidal conjugative transposon, termed CTnDOT, is particularly important for the ability of this group of gut commensals to spread erythromycin and tetracycline resistance. Interestingly, transfer of CTnDOT is triggered by exposure to low levels of tetracycline [66], thereby directly linking antibiotic use and the spread of antibiotic resistance determinants. In addition to the strictly anaerobic gut commensals discussed above, facultatively anaerobic bacteria, in particular the lactic acid bacteria (enterococci, streptococci and lactobacilli), appear to be important conduits of horizontal gene transfer in the intestinal tract [67]. Enterococci appear to be exceptionally well suited to function as ‘drug resistance gene traffickers’ in the human gut [68]. In addition, Enterobacteriaceae can readily exchange plasmids encoding antibiotic resistance and virulence genes during colonization of the intestinal tract [69,70].

The interaction between the human host and the microbiota and its effect on conjugative gene transfer in the gut deserves further attention as there appears to be a role for the mammalian host in determining the extent of horizontal gene transfer in the microbiota as human intestinal epithelial cells can produce a proteinaceous compound that can lower conjugational transfer of an antibiotic resistance plasmid between *E. coli* strains. [71]. By contrast, inflammation of the gut boosts conjugative gene transfer between pathogenic and commensal *E. coli* strains [72].

Phages are present in numbers that equal those of bacteria in the intestinal tract [73–75] and are hypothesized to importantly shape the composition of the human gut microbiota [76]. Analyses of metagenomic sequencing datasets have further expanded our understanding of the diversity and abundance of bacteriophages in the gut microbiota. Integrated prophages, which can carry antibiotic resistance genes, frequently enter a lytic cycle in the gut and could thereby transfer antibiotic resistance genes between gut bacteria [77]. Supplementing these findings, a recent study using quantitative PCR on phage DNA that was isolated from faeces of 80 healthy humans found that more than 70% of samples were positive for an antibiotic resistance gene, with the β-lactamases *bla*_TEM_ and *bla*_CTX-M−1_ and the quinolone resistance gene *qnrA* being most abundant [78]. Experimental evidence for the role of bacteriophages in the horizontal transfer of resistance genes has been obtained in a study where mice were treated with the β-lactam antibiotic ampicillin or the fluoroquinolone ciprofloxacin [79]. Both antibiotic treatments led to an increase of antibiotic resistance genes in the phage metagenome. Remarkably, when phages from antibiotic-treated mice were isolated and used to infect an aerobic culture of the microbiota of non-treated mice *ex vivo*, the frequency of resistant isolates was significantly higher compared with the culture that was infected with phages isolated from the microbiota of untreated mice. These data show that phage transduction can contribute importantly to the expansion of the gut resistome during antibiotic therapy [79].

## 5. Discussion and future perspectives

Antibiotic resistance genes are widely distributed in the environment and the studies that are discussed in this review have provided convincing evidence that the gut microbiota form a large reservoir for antibiotic resistance genes. Conjugative transfer of plasmids and transposons, and transduction are two important mechanisms by which antibiotic resistance genes can spread through the microbiota. However, there remain many open questions on the relative contribution of the gut resistome in donating antibiotic resistance genes to opportunistic pathogens.

The macrolide resistance genes *ermB*, *ermF* and *ermG* and the tetracycline resistance genes *tetM* and *tetQ* can spread among phylogenetically diverse Gram-negative and Gram-positive gut bacteria [60], but these may be exceptions as many of the resistance genes that are detected in gut commensals do not occur in opportunistic pathogens. Indeed, resistance genes from gut commensals appear to mostly spread to related bacteria. For example, the β-lactamase *cblA* is frequently present in *Bacteroides* and is one of the most abundant resistance genes in the microbiota of healthy individuals and patients [38,39]. However, this gene does not appear to transfer to opportunistic pathogens, like the Enterobacteriaceae, even though functional metagenomic selections have shown that this gene confers β-lactam resistance to *E. coli* [32,41]*.* The observation that the gut microbiota of two healthy volunteers contained nine β-lactamase sequence families (eight of which have highest identity to genes in *Bacteroides*) that had not previously been shown to occur in opportunistic pathogens [41], also indicates that major barriers exist for the transfer of resistance genes between anaerobic gut commensals (in particular, *Bacteroide*s) and Gram-negative facultative anaerobic opportunistic pathogens, like the Enterobacteriaceae. Conjugative transfer of resistance genes from *Bacteroides* to *E. coli* is possible under laboratory conditions [20,21], but, apparently, the conditions in the intestinal tract preclude the efficient mobilization and transfer of antibiotic resistance genes from Bacteroidetes to Enterobacteriaceae. The bacteroidal β-lactamases are therefore unlikely to contribute importantly to the burden of antibiotic resistance among Enterobacteriaceae.

It is less clear to what extent gut commensals from the phylum Firmicutes can transfer their resistance genes to Gram-positive, gut-dwelling opportunistic pathogens, such as enterococci and streptococci. A lack of data on the plasmids and conjugative transposons that are carried by the strictly anaerobic Gram-positive bacteria in the gut complicates the assessment of the transferability of these genes. However, conjugative transposons of the Tn*916*/Tn*1545* family (carrying tetracycline, macrolide and aminoglycoside resistance genes) are widely disseminated in clostridia, enterococci and streptococci, suggesting that resistance gene transfer occurs between these groups of bacteria [80]. Similarly, aminoglycoside resistance genes are carried by bacteria from the class Clostridia [32,81], even though anaerobic gut commensals are thought to be intrinsically resistant to aminoglycosides [82]. The role (if any) of these genes in aminoglycoside resistance in Clostridia is therefore unclear. Nevertheless, the aminoglycoside resistance genes from Clostridia are also found in enterococci and Enterobacteriaceae [83], again suggesting that Gram-positive gut commensals serve as hubs for the transfer of resistance genes. Arguably, the *vanB* transposon is clinically the most relevant antibiotic resistance determinant that can be acquired from the microbiota. Clinical *E. faecium* strains are able to acquire the *vanB* transposon, and gain resistance to vancomycin at a minimal fitness cost [84]. The recent, and largely unexplained, increase of *vanB-*positive *E. faecium* strains in Europe [85,86] may possibly be caused by a further spread of *vanB*-type transposons which are then repeatedly and independently acquired by vancomycin-susceptible *E. faecium* isolates.

Currently, it is already feasible to use metagenomic sequencing as a diagnostic tool for the detection of pathogens in faecal samples in outbreak situations [87] and fluctuations of the resistome have been tracked in individual patients [32,42]. The routine implementation of metagenomic sequencing of the microbiota in clinical practice is some way off as costs for sequencing and analysis are still high. Perhaps more importantly, the diagnostic relevance of resistome profiling by sequencing is currently limited as it takes at least several days to perform sequencing and analyses. Future advances in DNA sequencing technologies are likely to facilitate high-throughput characterization of the resistome by metagenomic sequencing in hospitalized patients. The choice of antibiotics for therapy might then be guided, at least partially, by the composition and relative abundance of the antibiotic resistance gene reservoir in patients. To fully assess the risks that are associated with the selection for antibiotic resistance genes in gut commensals, further studies are needed to profile the antibiotic resistance genes from the gut microbiota, particularly with respect to their potential for horizontal gene transfer.
